# The Peptide Methionine Sulfoxide Reductase (MsrAB) of *Haemophilus influenzae* Repairs Oxidatively Damaged Outer Membrane and Periplasmic Proteins Involved in Nutrient Acquisition and Virulence

**DOI:** 10.3390/antiox11081557

**Published:** 2022-08-11

**Authors:** Marufa Nasreen, Remya Purushothaman Nair, Alastair G. McEwan, Ulrike Kappler

**Affiliations:** School of Chemistry and Molecular Biosciences, Australian Infectious Diseases Research Centre, The University of Queensland, St. Lucia, QLD 4072, Australia

**Keywords:** *Haemophilus influenzae*, methionine sulfoxide reductase, oxidative damage, biochemical reconstitution, extracellular proteins

## Abstract

Sulfoxide-damage repair mechanisms are emerging as essential for the virulence of bacterial pathogens, and in the human respiratory pathogen *Haemophilus influenzae* the periplasmic MsrAB peptide methionine sulfoxide reductase is necessary for resistance to reactive chlorine species such as hypochlorite. Additionally, this enzyme has a role in modulating the host immune response to infection. Here, we have analysed the enzymatic properties of MsrAB, which revealed that both domains of the protein are catalytically active, with the turnover number of the MsrA domain being 50% greater than that for the MsrB domain. MsrAB was active with small molecular sulfoxides as well as oxidised calmodulin, and maximal activity was observed at 30°C, a temperature close to that found in the natural niche of *H. influenzae*, the nasopharynx. Analyses of differential methionine oxidation identified 29 outer membrane and periplasmic proteins that are likely substrates for MsrAB. These included the LldD lactate dehydrogenase and the lipoprotein eP4 that is involved in NAD and hemin metabolism in *H. influenzae*. Subsequent experiments showed that *H. influenzae* MsrAB can repair oxidative damage to methionines in purified eP4 with up to 100% efficiency. Our work links MsrAB to the maintenance of different adhesins and essential metabolic processes in the *H. influenzae*, such as NAD metabolism and access to L-lactate, which is a key growth substrate for *H. influenzae* during infection.

## 1. Introduction

The pivotal role of methionine sulfoxide reductases in protecting living cells from oxidative stress has long been recognized; however, the role of these enzymes in enhancing the survival of bacterial pathogens in the host is still not fully understood [1,2,3,4]. Bacterial pathogens inhabit an environment that promotes oxidative stress, in which enzymes of the host innate immune response such as NADPH oxidase, superoxide dismutase and myeloperoxidase produce superoxide radicals, hydrogen peroxide and hypochlorite, respectively [5]. These reactive oxygen and chlorine species (ROS, RCS) cause damage to DNA, lipids and proteins, and the sulfur-containing amino acids cysteine and methionine are particularly prone to oxidation [1,5].

Oxidation of methionine residues can lead to a loss of biological function by disrupting protein structure, and methionine sulfoxide reductases are required to reverse oxidative damage to free and protein-bound methionine residues [1,2]. The cytoplasmic peptide-methionine sulfoxide reductases MsrA and MsrB that use a thiol-based redox relay for catalysis and reduce the *S-* and *R-*stereoisomers of methionine sulfoxide (MetSO), respectively, are particularly well studied [2,3,6]. However, periplasmic or outer-membrane-associated methionine sulfoxide reductases have recently been shown to play a pivotal role in protecting various bacterial pathogens from exogenous oxidative stress. MsrA/B-related enzymes have been identified in *Streptococcus pneumoniae*, *Neisseria* sp., *Helicobacter* and *Fusobacterium* [7,8,9,10,11], and in all cases were fusion proteins containing both an MsrA and an MsrB domain, some of which were anchored to the cell membrane. In these pathogens, MsrAB fusion proteins are required for full virulence, with an absence of MsrAB reducing disease severity [8,9,12]. However, while the structural and kinetic properties of many of these enzymes have been reported, no natural substrates have been identified to date.

In addition to these MsrA/B-related enzymes, a mononuclear molybdenum enzyme called MsrP has been identified in *E. coli*, *Salmonella typhimurium* and some other bacteria as essential for maintaining the function and integrity of proteins in the bacterial cell envelope [13,14,15]. Unlike MsrAB-type enzymes, MsrP obtains reducing equivalents from a membrane-bound cytochrome subunit, MsrQ, and can reduce both *S-* and *R-*MetSO stereoisomers. MsrP was shown to be under the control of the hypochlorite-inducible response regulator YedW in *E. coli* and was expressed highly in cultures challenged with hypochlorite [13]. MsrP is also one of the rare methionine sulfoxide reductases for which detailed information on native protein substrates is available. In addition to oxidised calmodulin, a model, non-physiological substrate, MsrP was proposed to repair oxidative damage to at least 20 *E. coli* proteins, including the periplasmic chaperone SurA and the Pal lipoprotein [13].

We have recently characterized three enzymes involved in MetSO reduction in the *H. influenzae* periplasm, the molybdenum enzymes DmsABC and MtsZ and the MsrAB methionine sulfoxide reductase that contains both an MsrA and MsrB domain [16,17,18,19]. Both of the molybdenum-containing enzymes were able to reduce free MetSO; however, expression levels of DmsABC were significantly lower than for MtsZ, which appears to be the major molybdenum-dependent methionine sulfoxide reductase in *H. influenzae* strain Hi2019 [17,19].

Expression of both *mtsZ* and *msrAB* was induced by exposure of the bacteria to hypochlorite and is controlled by an extracytoplasmic function sigma factor, RpoE2, that is conserved in *H. influenzae* strains. In keeping with this observation, MsrAB is required for the hypochlorite resistance of *H. influenzae* [16,18]. MsrAB was also involved in host immune response modulation, where in response to infections with an *H. influenzae msrAB* strain, higher levels of expression of antimicrobial peptides were observed, while expression of the antiapoptotic XIAP protein was reduced [18].

Here we have characterised the enzymatic properties of *H. influenzae* MsrAB and identified several natural protein substrates of the enzyme that reveal a physiological role for MsrAB in maintaining H. influenzae adhesins and enzymes involved in nutrient acquisition.

## 2. Materials and Methods

### 2.1. Media and Bacterial Growth Conditions for E. coli and Haemophilus influenzae

*E. coli* DH5α (ThermoFisher, Waltham, MA, USA), *E. coli* Rosetta (Novagen, Madison, WI, USA) and *E. coli* JM109λpir (New England Biolabs, Ispwich, MA, USA) and derivative strains were grown on Luria-Bertani (LB) medium or 2xYT broth at 37 °C for 16–18 h [20]. Competent *E. coli* were prepared using the method of [21]. Ampicillin (100 μg/mL), spectinomycin (50 μg/mL), chloramphenicol (60 μg/mL) and kanamycin (100 μg/mL) were added to culture media when needed.

Brain Heart Infusion broth BBL (BD Biosciences, Franklin Lakes, NJ, USA) or agar (Difco, Plymouth, MA, USA) or Chemically Defined Medium (CDM, RPMI 1640 supplemented with 7.5 mM inosine, 0.89 mM uracil, 24 mM sodium bicarbonate, 1 mM sodium pyruvate, 25 mM HEPES pH 7.5) [22] supplemented with hemin (10 μg/mL) and NAD (10 μg/mL) [23] were used to grow *H. influenzae* Hi2019 [24] and the Hi2019 *^msrAB^* [18] strain at 37 °C with 5% CO_2_ for 16–18 h. Kanamycin (10 μg/mL) was added to *H. influenzae* media when needed.

### 2.2. Standard Molecular and Biochemical Methods

Standard methods were used throughout [25]. Genomic DNA, plasmid and PCR products were isolated using the Genomic DNA mini kit (Life Technologies, Carlsbad, CA, USA), the Purelink Plasmid DNA miniprep kit and the Purelink Quick PCR Purification Kit (both Life Technologies, Carlsbad, CA, USA), respectively, accordingly to the manufacturer’s protocols. Restriction enzymes were from Life Technologies or NEB, T4 ligase from Promega (Madison, WI, USA) or Life Technologies. SDS-PAGE used the method of [26], desalting of protein samples used PD-10 columns (Cytiva, Marlborough, MA, USA), and BCA protein determinations (BCA-1 kit, Sigma Aldrich, St. Louis, MO, USA) were performed according to the manufacturer’s protocol.

### 2.3. Construction of Protein Expression Plasmids and Optimisation of Protein Expression Protocols

Protein expression used the pProexHtb (Invitrogen, Waltham, MA, USA) or pET22b-plus (Novagen, Madison, WI, USA) plasmids (P4 protein expression only). Insert fragments (*msrAB*, *etrx*, *trxR*, *trx* and the *hel* gene that encodes the eP4 Lipoproteins) were amplified from Hi2019 genomic DNA by PCR (Table 1) and purified, and restriction enzyme-based cloning was used to create the plasmids (Table 1). Plasmids were verified using PCR screening and protein expression tests using 5 mL LB and overnight incubation after adding 0.5 mM IPTG at the mid-exponential growth phase.

### 2.4. Protein Expression in E. coli

For expression of proteins, 100 mL LB medium in a 250 mL flask was supplemented with the required antibiotics and inoculated with the *E. coli* expression strain from a fresh overnight culture at OD_600nm_ = 0.07, followed by incubation at 37 °C with shaking at 200 rpm. Large-scale expression cultures used 1 L LB medium in 2.5 L flasks. When the cultures reached an OD_600nm_ of 0.6–0.8, IPTG was added, and the cultures were incubated further to allow protein production, as summarised for each protein in Table 2.

### 2.5. Preparation of Cell-Free Extracts

Small scale cell extracts were prepared either using Bug Buster Mastermix (Merck-Novagen, Madison, WI, USA), as per the manufacturer instructions, or a bead beater (Thomas Scientific, Swedesboro, NJ, U.SA; 0.1 mm glass beads, 2 mL screw-cap tube). Bead beater lysis of up to 1 mL cell suspension used six 30 s bead beating cycles at maximum speed. Lysates from both methods were centrifuged (22,308× *g*, RT, 5 min), and the supernatant (cell-free extract) was transferred to clean tubes. If required, insoluble components were resuspended in water. For large-scale preparations, protein expression cultures were harvested (2369× *g*, 4 °C, 30 min) and cell pellets resuspended in equilibration buffer (20 mM NaH_2_PO_4_, 0.5 M NaCl, 20 mM imidazole, pH 7.4) and lysed by three passages through a French press (Aminco) at 10,000 psi. Cell debris was removed by centrifugation (30,000× *g*, 30 min, 4 °C) and the supernatant collected for further experiments.

### 2.6. Periplasm Preparation

Periplasmic fractions were isolated using the method of [27]. Protein expression cultures (rMsrAB, 50 mL volume) were harvested by centrifugation (2369× *g*, 4 °C, 10 min), washed in 50 mL 1 × PBS and resuspended in 7.5 mL of ice-cold (4 °C) 20% sucrose/10 mM Tris-Cl pH 7.5 before the addition of 250 μL of 0.5 M EDTA, pH 8.0. Samples were incubated on ice for 10 min, followed by centrifugation at 2369× *g* at 4 °C for 10 min. Cell pellets were immediately resuspended in 5 mL ice-cold water and periplasm and protoplasts separated by centrifugation (2369× *g*, 4 °C, 10 min). The supernatant (periplasmic fraction) was removed to a clean tube, while the protoplast pellets were lysed using a bead beater. Malate dehydrogenase assays were used to assess the quality of the preparations, with activities in periplasmic fractions required to be at least 20-times lower than in the cytoplasmic extract. Malate dehydrogenase assays were performed as in [28]. Each assay (1 mL) contained 8 μL of 25 mM oxaloacetate, 20 μL of 10 mM NADH and 5–25 μL CFE or periplasmic extracts in 100 mM potassium phosphate buffer (pH 7.5), and consumption of NADH was monitored at 340 nm. Malate dehydrogenase activity was calculated in units/mg using the NADH extinction coefficient (6.22 mM^−1^ cm^−1^).

### 2.7. Purification of Histidine-Tagged Proteins

Purification of histidine-tagged proteins used His-trap columns (5 mL column volume (CV), Cytiva, Marlborough, MA, USA) and an AKTA FPLC System with UNICORN V3.10 control software (Cytiva, Marlborough, MA, USA). All purifications were carried out at 4 °C with degassed and filtered (pore size: 0.4 μM) buffers. Columns were equilibrated using buffer A (20 mM potassium phosphate, 0.5 M NaCl, 20 mM imidazole, pH 7.4) and eluted using buffer B (20 mM potassium phosphate, 0.5 M NaCl, 500 mM imidazole, pH 7.4) and a 10 CV gradient from 20 mM to 500 mM imidazole. Purified recombinant Thioredoxin (Trx) Reductase had a yellow colour, characteristic of the FAD cofactor found in these enzymes.

### 2.8. Enzyme Assays

All enzyme assays used a Cary 60 (Agilent Technologies, Santa Clara, CA, USA) equipped with a thermostated water bath. Reactions containing NAD(P)H/NAD(P) were monitored at 340 nm using extinction coefficient: 6.22 mM^−1^ cm^−1^. Unless otherwise stated, assays were carried out a 37 °C.

### 2.9. Thioredoxin Reductase (TrxR) Activity Assay

One millilitre assays contained 50 mM K_2_HPO_4_ buffer, pH 8.0 with 1% ethanol, 1 mM EDTA, 3 mM 5,5′-dithiobis (2-nitrobenzoate) (DTNB), 0.2 mM of either NADPH or NADH and 2 μM TrxR. DTNB-related absorbance changes were monitored at 412 nm [29], and activity was calculated using an extinction coefficient of 13.6 mM^−1^ cm^−1^.

### 2.10. Thioredoxin Activity Assay

The thioredoxin catalysed reduction of insulin disulfide bonds that results in insulin precipitation [30] was used to assess the reactivity of purified thioredoxins. Each reaction contained 3 μM purified thioredoxin, 1 mM EDTA, 0.017 μM insulin and 1, 3 or 5 mM DTT in 0.1 M K_2_HPO_4_ buffer, pH 8.0. A sample without the addition of thioredoxin was used as a control, and reactions were monitored at 650 nm for up to 120 min.

### 2.11. TrxR Thioredoxin Redox Relay Activity

In this assay, insulin disulfide bonds were reduced by thioredoxins, while oxidised thioredoxins were re-reduced by TrxR with NADPH as the TrxR reductant. Reactions contained 50 mM K_2_HPO_4_ buffer, pH 8.0, 6 μM thioredoxin, 3 μM thioredoxin reductase, 0.2 mM NADPH and 0.017 μM insulin; NADPH consumption was monitored at 340 nm [31].

### 2.12. MsrAB Assays—DTNB-Based Colourimetric Activity Assay

This endpoint assay measures the ability of MsrAB to reduce MetSO in the presence of DTT as the reductant [32]. Reactions (100 μL volume) contained 10 mM MgCl_2_, 30 mM KCl, 25 mM Tris-Cl pH 7.5, 100 μM DTT and 250 μM *D/L*-Met-SO and were incubated for 30 min at 37 °C before the addition of an equal volume of DTNB to a final concentration of 1 mM and further incubation for 10 min at 37 °C. Absorbances were read at 412 nm; reactions without enzyme were used as the blank.

### 2.13. NADPH-Dependent MsrAB Activity Assay

Methyl-p-tolyl sulfoxide (*S/R-*, *S-*, *R-* MPTS, all Sigma Aldrich, St. Louis, MO, USA) was used as a model artificial substrate in an NADPH-depended MsrAB assay [33]. The optimised assay contained 10 mM sulfoxide substrate, 2 μM rMsrAB, 20 μM rTrx, 5 μM rTrxR and 0.2 mM NADPH in 50 mM sodium phosphate buffer, pH 7.5, and was carried out at 37 °C. Consumption of NADPH was monitored spectrophotometrically at 340 nm. During assay optimisation, ratios of rTrx to rTrxR of 1:1 to 6:1 were tested, and rTrx concentrations were optimised using concentrations between 5 and 30 μM.

To determine kinetic parameters, 0.5–20 mM *S/R-*, 1–20 mM *S-* and 1–15 mM *R-*MPTS were used. K_M_app_ and V_max_app_ values were determined by nonlinear regression using the Michaelis–Menten equation using Prism 8.2.0 (GraphPad, San Diego, CA, USA). MsrAB activity was also tested at different pH values (pH 5–10) using a combined buffer system of 20 mM Tris-Cl/20 mM Glycine/50 mM sodium phosphate. Temperature dependence of the MsrAB reaction was determined at temperatures in the range of 15–55 °C and pH 7.5.

Where indicated, purified MsrAB (2 μM) was pre-treated with 1 mM H_2_O_2_, 50 μM HOCl, 2 mM NCT or 50 μM paraquat for an hour at room temperature, followed by rebuffering into 20 mM Tris-Cl pH 8.0 using PD-10 columns (Cytiva, Marlborough, MA, USA).

For detection of MsrAB activity in *H. influenzae* crude extracts, *H. influenzae* cultures growing at mid exponential phase were treated with 200 μM HOCl for 60 min before harvesting. Crude extracts were prepared as set out above, and 50 μL containing up to 480 μg of protein was used in each assay instead of purified rMsrAB.

### 2.14. MsrAB-Based Repair of Oxidised Proteins

In some NADPH-based MsrAB assays, MPTS was replaced by either oxidised calmodulin or oxidised, purified *H. influenzae* eP4. Calmodulin was oxidised as described in [19], while oxidised eP4 was produced using treatment with 200 μM HOCl for 60 min, followed by removal of HOCl using a PD10-column (Cytiva, Marlborough, MA, USA). Assays contained 2 μM MsrAB, 20 μM thioredoxin, 5 μM Thioredoxin reductase, 400 μM NADPH and either 4 μM oxidised calmodulin or 3.3–6.6 μM oxidised eP4 in 50 mM sodium phosphate buffer, pH 7.5 (250 μL volume), and were incubated at 37 °C for 2 h. Calmodulin repair assays were purified using a His-Gravitrap column to remove His-tagged rTrx that has a molecular mass similar to that of calmodulin. The flowthrough from the Gravitrap column was collected and concentrated before loading 30 μL of sample onto a 17.5% SDS-PAGE gel. For eP4, repair was assessed using mass spectrometry and the NADPH reduction rate during the incubation; eP4 oxidation and reduction could not be visualised on a gel as its molecular mass is similar to rMsrAB (untagged) and rTrxR (6xHis-tagged).

### 2.15. Assessment of Trx Reduction State

Assays (1 mL) contained 0.1 mM DTNB and 10–20 μM (final concentration, max volume added 100 μL) proteins in 20 mM Tris-HCl, pH 8.0. Absorbance readings at 412 nm were taken. The effective concentration range of thiol detectable with this assay was in the range of 1–150 μM thiol based on calibration using reduced glutathione and cysteine.

### 2.16. H. influenzae Samples for Proteome Analyses

*H. influenzae* Hi2019 wildtype and *msrAB* were grown using 150 mL CDM in a 250 mL flask under microaerobic conditions with a starting OD_600nm_ of 0.08. When the OD reached 0.6–0.7, either 50 μL of 0.64 M HOCl (final conc.: 200 μM) or 50 μL of sterile water were added, followed by incubation for a further 60 min. Ten millilitres of each culture were harvested, and cell pellets were stored at −80 °C. Proteome data was collected at the Metabolomics Australia, Qld, node using label-free quantitation. Peptide data from three biological replicates were filtered to exclude all cytoplasmic proteins. Oxidation of methionines was then assessed for methionine-containing peptides in all three biological replicates, and oxidation levels for individual proteins were compared between the wildtype and *msrAB* strain.

### 2.17. Outer Membrane Protein Isolation

*H. influenzae* cultures (20 mL) for Outer Membrane Protein isolation were grown under microaerobic conditions to an OD_600_ of 0.6–0.7 before 200 μM HOCl (final concentration) were added. Cultures were incubated for another hour, and outer membrane proteins were isolated as described in [34] using sodium *N*-lauroyl-sarcosine.

### 2.18. MS/MS Sample Preparation

Samples for MS/MS analyses were essentially prepared as in [35]. Dithiothreitol (DTT) (5 mM final concentration) was added to protein samples (50 μg) before incubation at 56 °C for 30 min. Samples were cooled to room temperature, iodoacetamide (IAA) (25 mM final concentration) was added, followed by incubation for 30 min in the dark and quenching of the IAA using 5 mM DTT. Trypsin/Lys C (Promega) was added in a 50:1 ratio (protein/trypsin), and the proteins were digested at 37 °C for 16 h. Samples were desalted using ZipTips (Millipore, Burlington, VT, USA) before separation using reversed-phase chromatography on a Shimadzu Prominence nanoLC system. Using a flow rate of 30 μL/min, samples were desalted on an Agilent C18 trap (0.3 × 5 mm, 5 μm) for 3 min, followed by separation on a Vydac Everest C18 (300 A, 5 μm, 150 mm × 150 μm) column at a flow rate of 1 μL/min. A gradient of 5–35% buffer B over 45 min, where buffer A = 1% ACN/0.1% FA and buffer B = 80% ACN/0.1% FA, was used to separate peptides. Using a Nanospray III interface, eluted peptides were directly analysed on a TripleTof 5600 instrument (ABSciex, Framingham, MA, USA). Gas and voltage settings were adjusted as required. MS TOF scan across *m*/*z* 350–1800 was performed for 0.5 s, followed by information-dependent acquisition of the top 20 peptides across *m*/*z* 40–1800 (0.05 s per spectra).

### 2.19. Preparation of Whole-Cell Samples for Shotgun Proteomics

Cell pellets were lysed in 25 μL SDS solubilisation buffer (5% SDS, 50 mM ammonium bicarbonate (ABC) pH 7.55), and disulfide bonds were reduced by adding 20 mM dithiothreitol (DTT) and incubating for 30 min at 60 °C, followed by alkylation with 40 mM (final conc.) iodoacetamide at RT in the dark for 30 min. Then, 2.5 μL of 12% phosphoric acid was added, followed by 165 μL of S-Trap binding buffer (90% MeOH, 100 mM ABC, pH 7.1). The samples were loaded onto an S-Trap^TM^ Micro Spin Column (Protifi, Farmingdale, MA, USA) and centrifuged at 4000× g. The columns were washed with 3 × 150 μL S-Trap binding buffer before 1 μL of Lys-C (stock conc: 1 μg/μL) was added, and on-column digestion was allowed to proceed for 60 min at 37 °C. Peptides were eluted using both 40 μL 50 mM ABC by centrifugation, followed by 40 μL of 50% acetonitrile, 0.1% formic acid for hydrophobic peptides. The combined eluted peptides were dried in a speed vac at 45 °C and resuspended with water. Samples were separated and MS/MS data collected using a ThermoFisher (Waltham, MA, USA) Q-ExactiveHFx, data analysis used ThermoFisher Proteome discoverer software (version 2.4). FASTA protein sequences of all identified proteins were downloaded from NCBI and used in batch SignalP 4.0 [36] and TMHMM [37] analyses to identify periplasmic and outer membrane proteins. Peptide sequencing data was accessed for each of these proteins, filtered to exclude peptides that did not contain methionine residues, and levels of methionine oxidation were identified. Differential oxidation was assessed for all proteins, where in the Hi2019 ^*msrAB*^ strain oxidation was observed in at least two of the three biological replicates.

### 2.20. Bioinformatic Analyses

To identify *H. influenzae* thioredoxins related to thioredoxins that had been previously used in MsrAB assays in other species, BLASTP [38] was used with either Thioredoxin from *E. coli* K12 or *Neisseria gonorrhoeae* FA1090 as search models. SignalP 4.0 [36] and ProtParam [39] were used to identify signal peptides and calculate the molecular mass, extinction coefficients and isoelectric point of proteins, respectively.

### 2.21. Statistical Analysis

Statistical analysis was performed in Prism 8.2.0 (GraphPad, San Diego, CA, USA). K_M app_ and V_max app_ values were determined by nonlinear regression using the Michaelis–Menten equation using Prism 8.2.0 (GraphPad, San Diego, CA, USA).

## 3. Results

### 3.1. H. influenzae MsrAB Maturation Requires Export to the Periplasm

**Figure 1:**
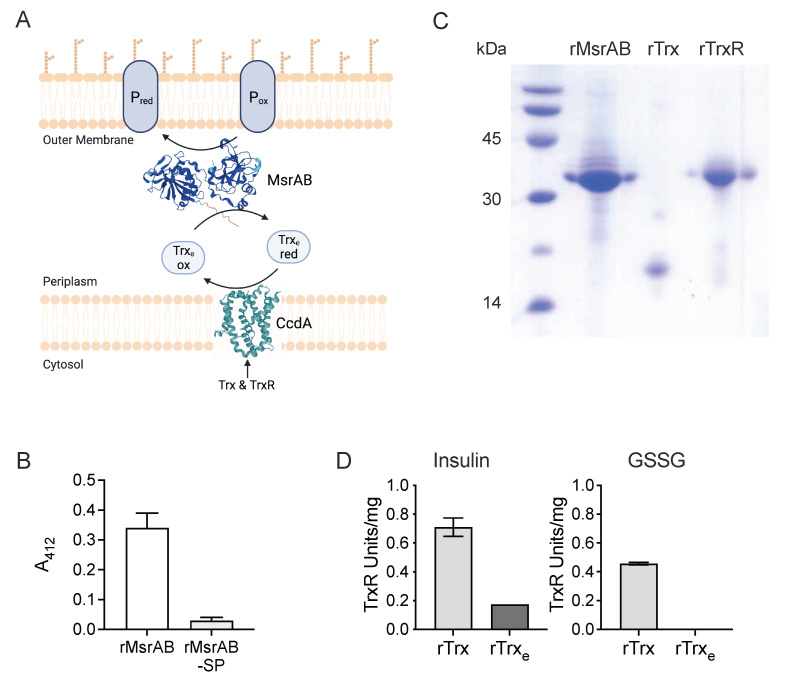
Properties of *H. influenzae* MsrAB and accessory redox proteins (**A**) Schematic representation of the MsrAB reaction cycle. P_ox_, P_red_—generic oxidised or reduced protein, TrxR—Thioredoxin Reductase, Trx—thioredoxin, Trx_e_—extracellular thioredoxin (**B**) Activity of rMsrAB and rMsrAB-Sp in an endpoint activity assay (**C**) Purified components of rMsrAB activity assay (**D**) Activity of the redox rMsrAB assay thioredoxin-based redox module using either insulin or oxidised glutathione (GSSG) as the artificial substrate. rMsrAB—recombinant methionine sulfoxide reductase activity, rTrxR—recombinant thioredoxin reductase, rTrx—recombinant thioredoxin, rTrx_e_—recombinant extracellular thioredoxin. Structural representations of MsrAB and CcdA (Panel A) are based on Alphafold database models. Panel A was generated using BioRender.

In vivo, the activity of Msr-type enzymes relies on complex, thiol-based redox relays where reactivation of the Msr enzymes after each reaction cycle is catalysed by thioredoxin. The oxidised thioredoxin is then re-reduced by a thioredoxin reductase (TrxR) or similar enzyme that most commonly links the reaction to the consumption of NAD(P)H (Figure 1). Since *H. influenzae* MsrAB is a periplasmic protein, the redox relay needs to be organized to enable transfer of reducing power from the cytoplasm to the periplasm. The *H. influenzae msrAB* operon encodes all components required for such a redox relay, namely a thioredoxin (redoxin family protein, WP_005631815.1, 15.1, Trx_e_) as well as a membrane-bound putative thiol-disulfide oxidoreductase, CcdA (cytochrome c biogenesis protein, WP_005656594.1), that is related to proteins involved in cytochrome *c* biogenesis and likely acts as the in vivo reductase of the *H. influenzae* MsrAB system [18]. To establish a specific activity assay for *H. influenzae* MsrAB, we overexpressed both MsrAB and the cognate Trx_e_ in *E. coli*. Recombinant forms of two additional proteins, the cytoplasmic *H. influenzae* thioredoxin reductase (WP_005689328.1, TrxR) and a cytoplasmic thioredoxin (WP_005689970.1, Trx), were also produced as related proteins have been used in activity assays for the extracellular MsrAB proteins from *N. gonorrhoeae* and *S. pneumoniae* [8,40]. TrxR can replace CcdA as the final, NADPH-linked reductase, while Trx is the *H. influenzae* Hi2019 thioredoxin with the highest sequence similarity to thioredoxins used in assays for other MsrAB proteins (Tables S1 and S2).

In *H. influenzae*, MsrAB is a periplasmic protein that can also occur associated with the outer membrane (18). As periplasmic enzymes from *H. influenzae* can often be successfully overexpressed in the *E. coli* cytoplasm (19), we constructed two overexpression plasmids, pProex-HiMsrAB-sp that leads to the production of a recombinant 6xHis-tagged, cytoplasmic MsrAB protein (rMsrAB-sp, 40.99 kDa) and pProex-HiMsrAB that enables recovery of untagged, recombinant MsrAB (rMsrAB, 37.62 kDa) from the *E.coli* periplasm, indicating that the *H. influenzae* MsrAB signal peptide was able to direct export of rMsrAB to the *E. coli* periplasm (Table 1 and Table 2).

Use of the two purified rMsrAB proteins in a DTNB-based endpoint assay with D/L-MetSO as the substrate revealed that the periplasmic rMsrAB led to an A_412 nm_ change of 0.34 ± 0.05, relative to the control, while, unexpectedly, rMsrAB-sp was essentially inactive, with an A_412nm_ change of only ~0.03 ± 0.01 (Figure 1B). We cannot completely exclude that the 6xHis-tag may have impacted the activity of rMsrAB-sp; however, as the tag is fused to the N-terminal MsrA-domain, activity should still have been observed for the C-terminal MsrB-domain. These results suggest that *H. influenzae* MsrAB requires export to the periplasm for maturation and activity, and rMsrAB was used for all further experiments.

### 3.2. The H. influenzae Extracellular Thioredoxin Is Sensitive to Air-Oxidation and Has Low Reducing Activity

We successfully expressed and purified the accessory, redox relay proteins rTrxR, rTrx_e_ and rTrx as 6xHis-tagged proteins and characterised them to establish their suitability for use in the Msr assay. As isolated, 20% rTrx_e_ and 70% rTrx were in the reduced state, indicating differences in the Trx redox potentials. We then assessed the ability of these thioredoxins to reduce insulin disulfide bonds with DTT as an external reductant [30]. Here, Trx_e_-containing reactions had significant lag times that may have been required for the initial reduction of this mostly oxidised thioredoxin and decreased from 40 min to 10 min with increasing DTT concentrations. Insulin reduction rates were moderate for Trx_e_, with 0.0089 Δ*A*_650nm_/min detected at 1 mM DTT and 0.031 Δ*A*_650nm_/min at 3 mM DTT (Figure S1). In contrast, rTrx insulin reduction rates exceeded those of rTrx_e_ 8.83- and 9.95-fold, respectively, at 1 mM and 2 mM DTT, and had short lag times of only 2–3 min. As a result of the higher activity of rTrx, insulin reduction rates plateaued at 0.1104 and 0.1154 Δ*A*_650nm_/min for 2 mM and 3 mM DTT (Figure S1).

### 3.3. rTrx-rTrxR Form a Suitable “Redox Module” for Electron Donation in an rMsrAB Assay

In keeping with previous descriptions of bacterial TrxRs [29,41,42], activity of the purified, recombinant *H. influenzae* thioredoxin reductase (rTrxR) in a DTNB-based assay was higher with NADPH (0.574 ± 0.043 U/mg) than with NADH (0.366 ± 0.012 U/mg) as an electron donor (Figure S1). We then tested the ability of rTrxR to drive thioredoxin-based reduction of insulin or oxidised glutathione (GSSG). With rTrx as the thioredoxin component, rTrxR activities were 0.71 ± 0.01 U/mg and 0.45 ± 0.01 U/mg with insulin and GSSG, respectively, while in assays containing rTrx_e_, activity was only detected with insulin (0.17 ± 0.001 U/mg) (Figure 1).

**Table 2:** Optimised protein expression conditions for recombinant proteins used in this study.

Plasmid	Protein	Protein Properties	*E. coli* Strain	Selective Markers (μg/mL)	IPTG (mM)	Post-Induction Temp.	Induction Time	Ref.
pProex-Htb MsrAB	rMsrAB	rMsrAB, expresses in *E. coli* periplasm, untagged	Rosetta	Amp 100, Cam 60	0.5	30 °C	2 h	This study
pProex-Htb-MsrAB-sp	rMsrAB-sp	rMsrAB-sp, MsrAB protein without the N-terminal signal peptide (aa 22–353), N-terminal 6xHis tag	Rosetta	Amp 100, Cam 60	0.5	30 °C	2 h	This study
pProexHtb Hi-eTrx	rTrx_e_	rTrx_e_*,* thioredoxin encoded in *msrAB* operon with N-terminal 6xHis tag	DH5α	Amp 100	0.1	37 °C	16–18 h	This study
pProexHtb-Hi-Trx	rTrx	rTrx, cytoplasmic thioredoxin with N-terminal 6xHis tag	DH5α	Amp 100	0.1	37 °C	16–18 h	This study
pProexHtb Hi-TrxR	rTrxR	rTrx-R, Thioredoxin reductase with N-terminal 6xHis tag	DH5α	Amp 100	0.5	37 °C	16–18 h	This study
pET_22_P4	eP4	eP4, P4 adhesin (aa 22–274), C-terminal 6xHis tag	BL21(DE3)	Amp 100	0.1	37 °C	2 h	This study; [43]

Amp—ampicillin, Cam—Chloramphenicol.

We then used the rTrxR-rTrx redox module to optimise an activity assay for rMsrAB using rTrxR to rTrx ratios of 1:1–1:6 and rTrx concentrations of up to 30 μM (Figure S1). The optimised assay contained 10 mM of a sulfoxide substrate, 2 μM rMsrAB, 20 μM rTrx, 5 μM rTrxR and 0.2 mM NADPH in 50 mM sodium phosphate buffer, pH 7.5, and was carried out at 37 °C.

### 3.4. rMsrAB Converts Both S- and R-Diastereoisomers of Sulfoxide Substrates with High Efficiency

**Figure 2:**
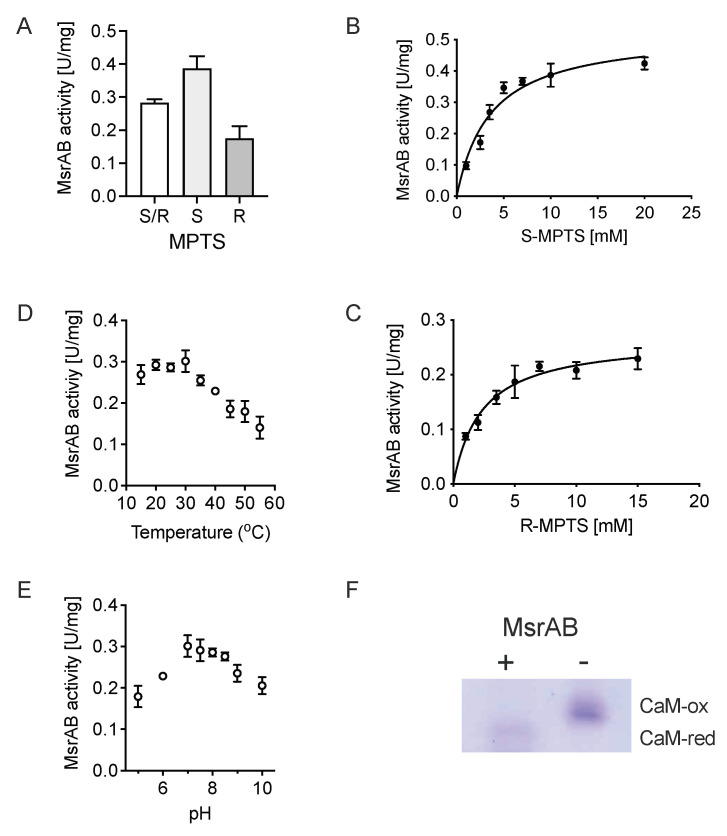
Catalytic activity of *H. influenzae* rMsrAB. (**A**): Stereospecificity of the rMsrAB reaction using *R/S*-, *R*- and *S*-Methyl-p-tolylsulfoxide (MPTS). (**B**): substrate concentration dependence of rMsrAB activity with *S*-MPTS. (**C**): substrate concentration dependence of rMsrAB activity with *R*-MPTS. Data was fit to the Michaelis–Menten Equation (see Table 3). (**D**): Temperature-dependence of rMsrAB activity. (**E**): pH-dependence of rMsrAB activity. (**F**): rMsrAB repair of oxidized calmodulin (CaM), ox—oxidized, red—reduced.

*H. influenzae* MsrAB contains both an MsrA and an MsrB domain that should be able to convert S- and R-sulfoxide stereoisomers, respectively. Using racemic R/S-Methyl-p-tolyl sulfoxide (*R/S-*MPTS), rMsrAB activity was 0.28 ± 0.01 U/mg, and the enzyme was also active with both *S-*MPTS (0.38 ± 0.03 U/mg) and *R-*MPTS (0.17 ± 0.03 U/mg). This shows that both catalytic domains are active, and that the MsrB domain has a lower activity than the MsrA domain (Figure 2). This apparent difference in the activity of the domains was largely driven by differences in the turnover rates of the two domains, which had *k*_cat_app_ values for *S-*MPTS (MsrA-domain) and *R-*MPTS (MsrB-domain) of 0.312 ± 0.12 s^−1^ and 0.156 ± 0.06 s^−1^, with associated *K*_M_app_ values of 3.62 ± 0.55 mM and 2.31 ± 0.35 mM, respectively (Figure 2, Table 3, Figure S2). The activity of rMsrAB only showed minor pH dependence and plateaued between pH 7.0 and pH 8.5 at ~ 0.29 ± 0.02 U/mg, with slight decreases toward pH 5 and pH 10, respectively (Figure 2). Unexpectedly, rMsrAB activity was highest at 30 °C and below, but decreased at higher temperatures, which was unexpected given the demonstrated role of MsrAB in *H. influenzae* virulence, where temperatures are usually around 37 °C (Figure 2) [18].

### 3.5. H. influenzae MsrAB Can Repair MetSO Damage in Proteins

The proposed natural function of MsrAB is the repair of oxidative damage in *H. influenzae* proteins, and we used oxidised calmodulin to determine whether rMsrAB is able to reduce methionine sulfoxide residues in proteins. Recombinant *E. coli* MsrP, a molybdenum enzyme with a known ability to repair oxidized proteins, was used as a control, and changes in the oxidation state of calmodulin were detected as differences in electrophoretic mobility, as found previously [13,19]. Following incubation with 4 μM oxidised calmodulin for 120 min, similar electrophoretic mobility differences compared to the oxidised calmodulin were observed for both the MsrP control and the rMsrAB assay, demonstrating that rMsrAB can reduce protein-bound methionine sulfoxides (Figure 2 and Figure S1).

### 3.6. MsrAB Is Sensitive to Inactivation by Reactive Oxygen and Chlorine Species

**Figure 3:**
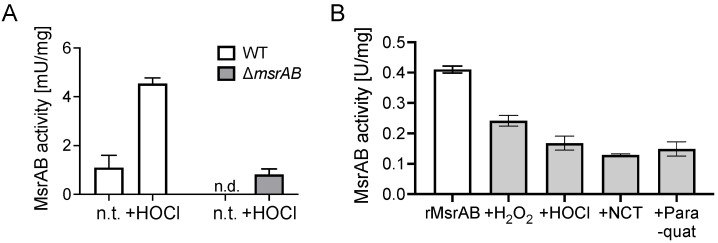
Effects of oxidative stress agents on rMsrAB activity. (**A**): MsrAB activity in Hi2019 wildtype and *msrAB* strains during microaerobic growth with or without exposure to hypochlorite. (**B**): Effect of exposure to ROS or RCS on activity of purified rMsrAB. n.t.—not treated; n.d.—not detected. NCT—N-Chlorotaurine.

The thioredoxin-based MsrAB assay could also be used to detect MsrAB activity in *H. influenzae* strains Hi2019 cell extracts where 4.5 ± 0.2 mU/mg of activity were present following treatment with 200 μM HOCl, while in untreated samples, activity was essentially absent (Figure 3). This confirms our previous observation that exposure of *H. influenzae* to HOCl and other oxidizing reagents produced by the human immune system increases *msrAB* gene expression [18]. However, it also raises the question whether these ROS/RCS that occur at sites of infection could cause oxidative damage to MsrAB itself, as its catalytic mechanisms relies on functional thiol groups that are highly susceptible to ROS- and RCS-induced damage [1]. To test this, we exposed purified rMsrAB to hydrogen peroxide, paraquat, hypochlorite or N-Chlorotaurine and used the treated protein in activity assays. The ROS/RCS exposure reduced rMsrAB activity by between 42% (hydrogen peroxide) and 61% (N-Chlorotaurine), but none of the treatments completely abolished rMsrAB activity (Figure 3). This suggests that while essential for hypochlorite resistance, in vivo, MsrAB itself may also be inactivated by oxidative damage, which then raises the possibility that functional MsrAB proteins may be involved in repairing oxidative damage to other MsrAB molecules.

### 3.7. Identification of Putative H. influenzae MsrAB Target Proteins

The bacterial cell envelope is the first point of contact for external oxidants such as HOCl, and MsrAB is likely to be involved in preventing damage to proteins found in the *H. influenzae* cell envelope [18]. To identify potential substrates for *H. influenzae* MsrAB, we analysed the oxidation state of proteins isolated from cultures of Hi2019^WT^ and the Hi2019^Δ*msrAB*^ strain that lacks a functional MsrAB protein [18] after treatment with 200 μM HOCl for 60 min. Unexpectedly, the overall levels of methionine oxidation were very similar in treated and untreated proteome samples, and for the WT strain even slightly decreased from 5.7% of Met-containing peptides to 4.3% following HOCl exposure. In comparison, in the Δ*msrAB* mutant strain, the oxidation levels increased slightly from 4.9% to 5.2%.

We then specifically analysed MetSO formation in periplasmic and outer membrane proteins to identify potential MsrAB substrate proteins that would show increased MetSO damage in the Δ*msrAB* strain. This approach identified 23 proteins with increased oxidative damage in at least two of the three biological replicates (Table S3). These included key NTHi virulence factors such as protein D, the lipoprotein eP4 that is involved in NAD uptake and several ABC transporter substrate-binding proteins required for the uptake of putrescine, methionine, glucose/galactose, oligopeptides and C4 dicarboxylates (Table 4). To confirm our results, we also analysed differential protein oxidation in outer membrane protein preparations, which also identified the PotD putrescine substrate-binding protein, the PntA transhydrogenase, LldD lactate dehydrogenase and Protein D as likely MsrAB substrates, but also added another six putative MsrAB substrate proteins. In the outer membrane preparation, differential oxidative damage was also detected for a penicillin-binding protein, BamA, and the HMW adhesin (Tables S3 and S4). Taken together, these data show that a significant number of periplasmic and membrane proteins are damaged by oxidative stress in the Δ*msrAB* strain, suggesting that MsrAB is critical in the maintenance of the functionality of these essential virulence factors.

### 3.8. MsrAB Can Efficiently Repair Oxidised Lipoprotein eP4 from H Influenzae

**Figure 4:**
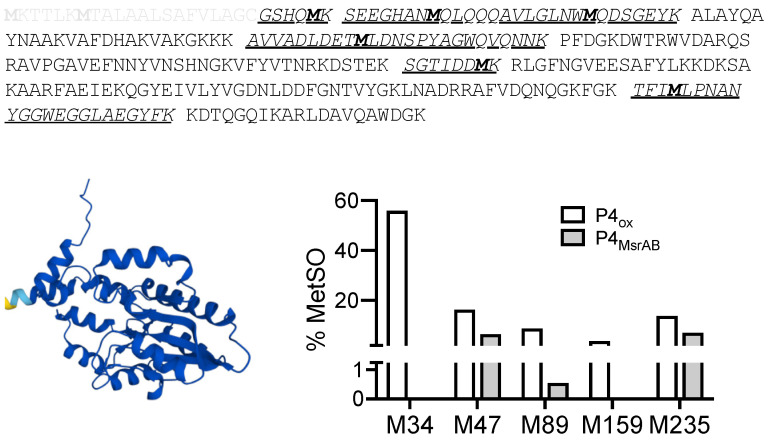
*H. influenzae* rMsrAB can repair oxidative damage to lipoprotein eP4. (**Top**): Amino acid sequence of lipoprotein eP4: bold—methionine residues with amino acid number in sequence; underlined—methionine containing peptides; grey font—eP4 lipoprotein signal peptide, not part of the expression construct. (**Bottom left**): Structural representation of eP4, based on Alphafold database structural model. (**Bottom right**): Methionine oxidation in oxidised P4 protein samples and following repair of oxidative damage with rMsrAB. M-Methionine.

One of the potential MsrAB substrate proteins, lipoprotein eP4, was selected for verification of rMsrAB activity towards native proteins. Lipoprotein eP4 was overexpressed as a soluble protein without the lipoprotein-signal, essentially as in [43], and oxidised for use in an MsrAB assay. In assays containing oxidised eP4, MsrAB activity levels between 0.13 U/mg and 0.34 U/mg were determined, with activity directly proportional to the amount of eP4_ox_ added (3.3 μM and 6.6 μM, respectively). To ascertain that eP4 MetSO-damage was repaired during the assay, assay mixtures were analysed by MS/MS and methionine oxidation levels in eP4 peptides compared to the oxidised eP4 used as the substrate. We detected five of the six methionine residues present in the recombinant eP4 (Figure 4), with initial methionine oxidation levels between 3.8% and 55.9% in the oxidised eP4 sample. Following incubation with rMsrAB in the repair assay, a significant reduction in methionine oxidation ranging from 48% (Met235) to 100% (Met34, Met159) was observed (Figure 4). This clearly demonstrates that rMsrAB is highly effective at repairing oxidative damage to eP4, validating it as a native substrate protein.

## 4. Discussion

Msr-type methionine sulfoxide reductases have long been known to protect living cells from oxidative damage to methionine residues in proteins, a critical process that maintains protein function [1,2,3,4]. However, detailed knowledge of the proteins that are substrates for Msr enzymes is lacking, and this limits an understanding of the impact of oxidative damage to protein Met residues on the molecular physiology of the cell. Only a handful of substrate proteins for Msr-type methionine sulfoxide reductases have been identified to date, and most of these proteins were substrates for cytoplasmic methionine sulfoxide reductases in *E. coli* and *H. pylori*, where the function of Msr proteins has been studied most extensively [3]. While a range of natural substrates were reported for the *E. coli* periplasmic methionine sulfoxide reductase, MsrP [13], this enzyme is a molybdenum-containing methionine sulfoxide reductase and is structurally and catalytically distinct from MsrAB. Native substrates for the extracellular, fused-domain MsrAB enzymes have, to the best of our knowledge, not been reported so far.

Here we have investigated the enzymatic properties of the periplasmic *H. influenzae* MsrAB enzyme. We have shown that this enzyme requires export to the periplasm for full maturation, and that both enzymatic domains are active in a thioredoxin-dependent assay that we developed. The kinetic parameters determined for rMsrAB using *R/S*-MPTS, *S-*MPTS or *R-*MPTS are most similar to those reported for other bifunctional MsrAB fusion proteins isolated from the bacterial pathogens *Neisseria meningitidis* and *Streptococcus pneumoniae* [40,44]. Notably, all three MsrAB fusion proteins had turnover numbers (*k*_cat_) below 1 s^−1^, and in kinetic assays, the MsrB domain that converts the *R-*MetSO had a lower turnover number than the MsrA domain in both *H. influenzae* and *S. pneumoniae* [44]. At the same time, the MsrB domain had a lower K_M_ value for small molecular sulfoxides, indicating that MsrB reaches maximal turnover at lower substrate concentrations than MsrA. Similar data have also been reported for the cytoplasmic *E. coli* MsrA and *Neisseria meningitidis* MsrB proteins during an extensive characterisation of the Msr reaction mechanism [45]; however, these intracellular enzymes had higher turnover numbers between 1.1 and 3.7 s^−1^. An increase in K_M___R/S-MetSO_ at pH 5.5 to 7 mM compared to 2.2 mM at pH 8.0 has been reported for the *N. meningitidis* MsrAB enzyme, and we propose that a similar change in kinetic parameters could explain the reduction in *H. influenzae* rMsrAB activity towards pH 5.

In addition to establishing the kinetic properties of *H. influenzae* rMsrAB, we also showed that this enzyme can repair MetSO-damage to methionine-rich model proteins and native *H. influenzae* proteins. Especially with the native substrate protein eP4, rMsrAB could reverse greater than 94% of the MetSO formation for three out of five eP4 methionine residues, and for repair of the remaining two methionine residues, efficiencies were 48% and 60%, respectively. This demonstrates the high effectiveness of rMsrAB protein repair. MsrAB itself could be partially inactivated following exposure to oxidative stress reagents, which opens up the possibility that in vivo *H. influenzae* MsrAB may also repair damaged MsrAB proteins and thereby maintain sulfoxide-repair capacity.

Our proteome analysis identified 29 putative MsrAB substrate proteins, including protein eP4, which is the first time that the targets for sulfoxide repair have been identified for an extracellular MsrAB methionine sulfoxide reductase. The 29 MsrAB substrate proteins fell into several broad categories that included transport proteins, enzymes and virulence factors and, lastly, several proteins of unknown function. Interestingly, the types of proteins identified as *E. coli* MsrP substrates also included a large number of transport proteins, as well as a smaller number of lipoproteins and chaperones. In *H. influenzae*, we identified 7 ABC transporter substrate-binding proteins with specificities for substrates as diverse as sialic acid, putrescine, C4-dicarboxylates, methionine or glucose. A subunit of the Sec protein export system, SecD, was shown to be a potential MsrAB substrate as well as a group of lipo- and membrane proteins that are known virulence factors, such as Protein D (GlpQ), opacity-associated proteins, the OmpA porin, the HU toxin-repeat protein, the HMW adhesin and an IgA1 protease domain-containing protein. Proteins with metabolic functions such as the LldD lactate dehydrogenase, which is essential for *H. influenzae* virulence and is also a known cargo of *H. influenzae* OMVs [46,47], the NapC subunit of nitrate reductase and a subunit of the PNT transhydrogenase were also identified as putative substrates for MsrAB.

The identification of the Lipoprotein eP4 as a substrate of MsrAB is particularly interesting in the context of metabolic function and the effects of an *msrAB* mutation on NTHi fitness. The eP4 protein is required for uptake or utilisation of two essential *H. influenzae* nutrients, hemin and NAD, and has been demonstrated to be required for growth in the presence of NAD^+^ but not nicotinamide mononucleotide [48,49,50]. Most recently, eP4 has been implicated in interactions with host cells and was reported to efficiently bind laminin and fibronectin [43], while an absence of eP4 reduced the survival of *H. influenzae* in a mouse otitis media model [43]. This makes eP4 a key virulence factor along with Protein D, Oap and HMW and demonstrates why rMsrAB is an essential part of the *H. influenzae* defence against oxidative stress.

In summary, this work provides the first detailed molecular insights into the physiological role of MsrAB in *Haemophilus influenzae*. The identification of native substrates for MsrAB links this enzyme to the maintenance of virulence factors and to key proteins involved in the acquisition and utilisation of essential growth substrates. While identifying and verifying native substrate proteins can be a complex process, this information is vital to determine the link between the biochemical specificity of peptide methionine sulfoxide reductases, the maintenance of protein function, and the protection of crucial physiological processes during oxidative stress in bacterial pathogens.

## Figures and Tables

**Table 1:** Plasmids and Primers.

Plasmid	*H. influenzae* 2019 Gene Loci	Plasmid Insert Generation	
		*Primer Name*	*Primer Sequences*
**pProex-Htb MsrAB**	C645_RS07025	Hi_msrAB_pPro_Bam_F	AAAA**GGATCC**ATGAAACTATCAAAAACATTTC
		Hi_msrAB_pPro_Hind_R	AAAA**AAGCTT**CTTATTTTTTAATGGATTGAATC
pProex-Htb-MsrAB-sp	C645_RS07025	Hi_msrAB-sp_pPro_BamHI_F	AAAA**GGATCC**ATACAAAATTCAACATCATCATC
		Hi_msrAB_pPro_Hind_R	AAAA**AAGCTT**CTTATTTTTTAATGGATTGAATC
pProexHtb Hi-eTrx	C645_RS08405	Hi_etrx_pPro_Bam_F	AAAA**GGATCC**CAAACTAATTTGGCAGATGT
		Hi_etrx_pPro_XbaI_R	AAAA**CTGCAG**CTTTTCATTATTTCCCT
pProexHtb Hi-TrxR	C645_RS07025	Hi_trxR_pPro_BamHI F	AAAA**GGATCC**TCAGATACCAAACACGCAAAAC
		Hi_trxR_pPro_Eco R	AAAA**GAATTC**TTAAAAGAGGGGAATTGGTTAG
pProexHtb-Hi-Trx	C645_RS00695	Hi_trx_pPro_BamHI_F	AAAA**GGATCC**AGCGAAGTATTACACATTAATGA
		Hi_trx_pPro_Eco_R	AAAA**GAATTC**CAACGATTAAATATGTTGGTTAA
pET_22_P4	C645_RS09250	HiP4_pET_Bam_F	AAAA**GGATCC**GGGTTCACACCAAATGAAATCAGAA
		HiP4_pET_SacI_R	AAAA**GAGCTC**GCTTTACCATCCCAAGCTTGTACTGC

**Table 3:** Kinetic properties of *H. influenzae* rMsrAB in thioredoxin/thioredoxin reductase-based assays using MPTS as the substrate.

Substrate	V_max_app_ (U/mg)	*k*_cat_app_ (s^−1^)	K_M _app_ (mM)	*k*_cat_/K_M_ (s^−1^ M^−1^)
*S/R*-MPTS	0.34 ± 0.01	0.204 ± 0.06	2.96 ± 0.44	68.9
*S*-MPTS	0.52 ± 0.02	0.312 ± 0.12	3.62 ± 0.55	86.1
*R*-MPTS	0.26 ± 0.01	0.156 ± 0.06	2.31 ± 0.35	67.5

**Table 4:** Differences in methionine oxidation in Hi2019^WT^ and Hi2019^Δ*msrAB*^ following treatment with HOCl.

			% Met Oxidation	
Accession	Protein Name	Peptides	WT	Δ*msrAB*
**Transport Proteins**				
WP_005651801.1	ABC transporter substrate-binding protein OppA	VAIAAASmWK	0	12.23 ± 4.45
		AmAESYAATDAEGR	0	5.43 ± 0.75
WP_005655633.1	galactose ABC transporter substrate-binding protein MglA	LLmNDSQNAQSIQNDQVDVLLSK	0	29.16 ± 5.89
		YDDNFmSLMR	0	20.83 ± 17.67
WP_005657776.1	C4-dicarboxylate ABC transporter substrate-binding protein DctP-like	AADDSMmYHK	42.22 ± 22.68	71.1 ± 7.69
		mIAETTQEAK	6.94 ± 1.49	22.5 ± 3.53
WP_005688477.1	putrescine/spermidine ABC transporter substrate-binding protein PotD	APLNmVFPK	0 *	10.1 ± 3.36
WP_012054840.1	sialic acid-binding protein SiaP	FGmNAGTSSNEYK	0 *	36.1 ± 12.72
WP_046067550.1	methionine ABC transporter substrate-binding protein MetQ	VGVmSGPEHQVAEIAAK	0 *	13.9 ± 10.01
WP_005650782.1	glycerol-3-phosphate transporter GlpT	FVMAGmSDR	0 *	50.2 ± 46.58
WP_005653411.1	preprotein translocase subunit SecD	NmLPADSEVKYDR	0 *	75 ± 35.35
**Enzymes and Virulence Factors**				
WP_005631652.1	NADP transhydrogenase subunit alpha PntA	VmSEEFNRR	0	100 ± 0
		mQNPELMK	0	26.78 ± 2.52
WP_005657875.1	alpha-hydroxy-acid oxidizing enzyme LldD	DmHSGMSGPYK	9.72 ± 8.67	33.2 ± 14.36
		MLALGADATmLGR	0 *	33.3 ± 0
WP_005687981.1	thiamine biosynthesis lipoprotein ApbE	TmGTTYHVK	0	41.66 ± 11.78
WP_005649107.1	cytochrome *c* NapC	LEmAQNEWAR	0	16.7 ± 28.86
WP_046067759.1	membrane protein OmpA	ANLKPQAQATLDSIYGEmSQVK	26.13 ± 1.61	41.66 ± 11.78
WP_046067581.1	opacity-associated protein OapA	ATAPVQPmKK	0 *	83.33 ± 23.57
WP_005647222.1	Outer membrane protein SlyB	mSQVNGAELVIK	0	33.34 ± 0
WP_005661229.1	glycerophosphoryl diester phosphodiesterase GlpQ/Protein D	IKTELLPQmGMDLK	0	46.7 ± 17.63
		ALAFAQQADYLEQDLAmTK	0	32.5 ± 10.61
WP_005661232.1	5′-nucleotidase, lipoprotein e P4 family	DSTEKAGTIDDmKR	0	50 ± 0
WP_046067826.1	Histone	ASEmKEAASEKASEMK	0	93.3 ± 11.54
		EAVSEKASEmKEAASEK	0	100 ± 0
		EAASEKASEmKEAASEK	0	61.1 ± 34.69
		EAASEKTSEmKEAVSEK	0	50 ± 0
		DAAANTmTEVK	0	21.36 ± 10.91
**Proteins of unknown function**				
WP_005656625.1	hypothetical protein	KPLNAEMAmTR	0	20 ± 0
WP_005668839.1	hypothetical protein	SSAQMAEmQTLPTITDK	0	23 ± 23.38
WP_005655950.1	hypothetical protein	NGmVEVQKNEDGTPK	0	61.1 ± 34.69
WP_005689200.1	membrane protein	NGEAYLmPK	47.77 ± 27.16	55.6 ± 9.62
WP_005658466.1	membrane protein	IVAPmQR	0	50.0 ± 0

* indicates values where two of three biological replicates showed no oxidation.

## Data Availability

Data is contained within the article or supplementary material.

## Supplementary Materials

The following supporting information can be downloaded at: https://www.mdpi.com/article/10.3390/antiox11081557/s1, Table S1: Properties of proteins with a Trx motif (CXXC) in Hi2019. Table S2: Thioredoxin-related proteins in *H. influenzae* 2019 that are close homologues to *E. coli* K12 and *N. gonorrhoeae* FA 1090 thioredoxins. Table S3: Mass spectrometry data—peptide sequencing data. Table S4: putative *H. influenzae* MsrAB substrate proteins identified using outer membrane preparations. Yellow—also differentially oxidized in whole cell proteome. Figure S1: Properties of accessory proteins and development of the rMsrAB assay. Figure S2: Changes of rMsrAB activity in thioredoxin-containing assays using *R/S*-MPTS as the substrate.
