# Supplementary material for: The Peptide Methionine Sulfoxide Reductase (MsrAB) of Haemophilus influenzae Repairs Oxidatively Damaged Outer Membrane and Periplasmic Proteins Involved in Nutrient Acquisition and Virulence
Source: Antioxidants (Basel). 2022 Aug 11;11(8):1557. doi: 10.3390/antiox11081557 (PMC9404787; doi:10.3390/antiox11081557)

Table S1: Properties of proteins with a Trx motif (CXXC) in Hi2019. Grey shaded indicated the Trx_e and Trx used in the study.

**Accession No**	**Protein name**	**Signal Peptide**	**No. of amino acids**	**Molecular mass mature proteins (Da)**	**Gene locus**	**pI**
WP_005689970.1	thioredoxin (Trx)	No	107	11640.5	C645_RS00695	4.85
WP_005688304.1	protein disulfide oxidoreductase (TlpA)	No	167	19104.1	C645_RS06420	9.06
WP_005689328.1	thioredoxin-disulfide reductase (TrxR)	No	318	34398.8	C645_RS06360	5.24
WP_042594435.1	thiol:disulfide interchange protein (DsbE)	Yes	181	20528.9	C645_RS05695	6.18
WP_046067689.1	thiol:disulfide interchange protein (DsbD)	Yes	579	62460.7	C645_RS05395	6.29
WP_005631815.1	Redoxin family protein (Trx_e)	Yes	156	15598.8	C645_RS08405	5.4

Table S2: Thioredoxin-related proteins in *H. influenzae* 2019 that are close homologues to *E. coli* K12 and *N. gonorrhoeae* FA 1090 thioredoxins.

**Accession No.**	***H. influenzae* protein annotation**	**Closest homologue in *E. coli* or *N. gonorrhoeae***	**Query cover**	**AA identity**	***H. influenzae* gene Locus**
WP_005689970.1	Thiol reductase thioredoxin	WP_001280776.1, Thioredoxin TrxA [*E. coli* MG1655]	96%	51.43%	C645_RS00695
WP_046067700.1	Thiol:disulfide interchange protein DsbE	WP_000824439.1, thiol:disulfide interchange protein DsbE [*E. coli* MG1655]	77%	44.06%	C645_RS05695
WP_005688304.1	protein disulfide oxidoreductase	WP_003687293.1, protein disulfide oxidoreductase [*N. gonorrhoeae* FA 1090]	98%	45.18%	C645_RS06420
WP_042594435.1	Thiol:disulfide interchange protein	WP_000824439.1, thiol:disulfide interchange protein DsbE [*E. coli* MG1655]	84%	57.96%	C645_RS06360
WP_005689328.1	Thioredoxin-disulfide reductase	WP_000537418.1, thioredoxin-disulfide reductase [*E. coli* MG1655]	98%	76.51%	C645_RS06915
WP_046067689.1	Thiol:disulfide interchange protein	WP_003696987.1 protein-disulfide reductase DsbD [*N. gonorrhoeae* FA 1090]	93%	34.95%	C645_RS05395
WP_005631815.1	hypothetical protein	WP_010951394.1, (Trx domain) Trifunctional thioredoxin/methionine sulfoxide reductase A/B protein [*N. gonorrhoeae* FA 1090]	78%	32.26%	C645_RS08405

Table S3: Mass spectrometry data -peptide sequencing data. Separate Excel spreadsheet.

Table S4: putative *H. influenzae* MsrAB substrate proteins identified using outer membrane preparations. Yellow– also differentially oxidized in whole cell proteome

			% MetSO	
**Accession**	**Protein name**	**Peptides**	**WT**	***ΔmsrAB***
**Membrane proteins**				
WP_046067692.1	Outer membrane protein assembly factor BamA	FEGNTVSADSTLRQEMRQQEGTWYNSQLVELGK	85	100
	Outer membrane protein assembly factor BamA	IVGNVGGMSAELEPLLSALHLNDTFR	47.1	64.52
WP_005656589.1	Membrane protein	NLLNLKPNEALNITFPHIMNVK	37	100
WP_046067790.1	HMW2B, OMP-85 required for HMW1A and HMW2A secretion	TAQLELQAVLDKIEPNKFDVVLPQQTITDGNVMFELVSK	30	100
**Others**				
WP_005658614.1	Hypothetical protein	QLTGAVVDAAYMYPFWQWVGGPWALVK	55	76
WP_046067852.1	Penicillin-binding protein	QNLDVILADPAQIQGMDVLALNATPNSR	31	73
WP_005667564.1	Elongation factor Tu	TTDVTGTIELPEGVEMVMPGDNIK	44	100
**Enzymes**				
WP_005661229.1	Glycerophosphoryl diester phosphodiesterase (Protein D)	YADGVGPGWYMLVNK	28	35
WP_005631652.1	PntA NAD(P) transhydrogenase subunit alpha	AQALDALSSMANISGYR	30	45
WP_005657875.1	LldDAlpha-hydroxy-acid oxidizing enzyme	MIISSASDYR	0	100
**Transporter**				
WP_005688477.1	Putrescine/spermidine ABC transporter substrate-binding protein	VIVSSLESNETMYAK	50	100

Figure S1: Properties of accessory proteins and development of the rMsrAB assay **A:** Activity of recombinant Thioredoxin reductase (rTrxR) with NADH or NADPH (substrate: DTNB). **B:** Insulin reduction by isolated rTrx and rTrxe. Activity is determined as an increase over time in the absorbance at 600 nm. **C:** rMsrAB assay with rTrxR and rTrx – effect of varying concentrations of rTrx on rMsrAB activity. Assay contained 10 mM R/S MPTS, 2 µM rMsrAB, varying amounts of purified rTrx, 5 µM rTrxR and 0.2 mM NADPH in 50 mM sodium phosphate buffer, pH 7.5 **D:** rMsrAB calmodulin repair assay including the MsrP control reactions.

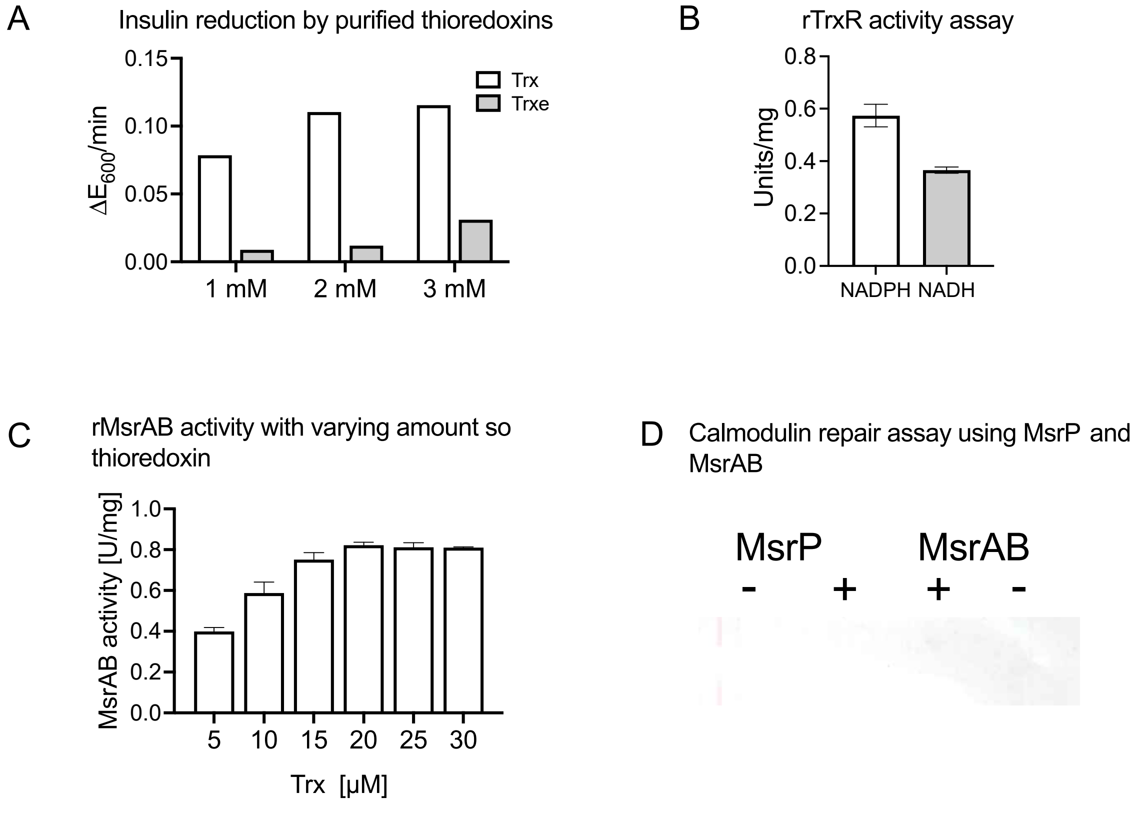

Figure S2: Changes of rMsrAB activity in thioredoxin-containing assays using *R/S*-MPTS

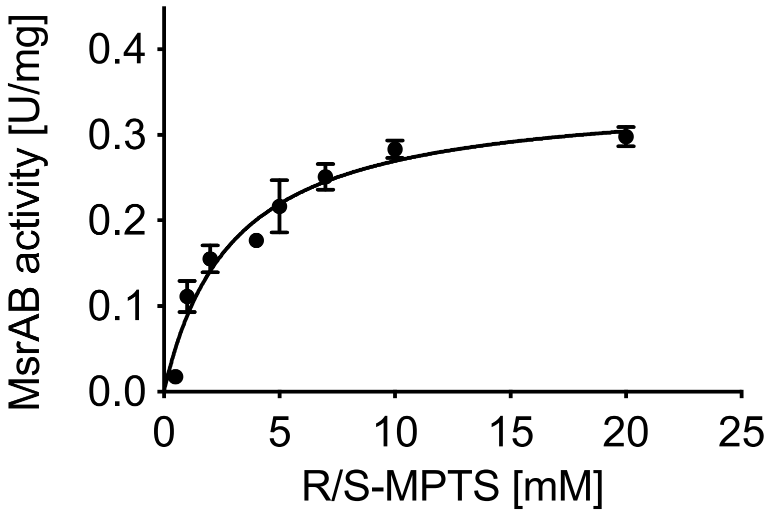

Supplement: Supplementary file 1 [file antioxidants-11-01557-s001.zip › Nasreen et al MsrAB Supp Data 060822.pdf]
